# Metabolomic Profiling Revealed Potential Biomarkers in Patients With Moyamoya Disease

**DOI:** 10.3389/fnins.2020.00308

**Published:** 2020-04-21

**Authors:** Chunmei Geng, Changmeng Cui, Yujin Guo, Changshui Wang, Jun Zhang, Wenxiu Han, Feng Jin, Dan Chen, Pei Jiang

**Affiliations:** ^1^Jining First People’s Hospital, Jining Medical University, Jining, China; ^2^Affiliated Hospital of Jining Medical University, Jining Medical University, Jining, China; ^3^Department of Clinical Translational Medicine, Jining Life Science Center, Jining, China; ^4^Department of Pharmacy, The First Affiliated Hospital of Zhengzhou University, Zhengzhou, China

**Keywords:** moyamoya disease, gas chromatography–mass spectrometry, biomarkers, serum, metabolomics

## Abstract

Metabolomics is increasingly used to observe metabolic patterns and disease-specific metabolic biomarkers. However, serum metabolite analysis of moyamoya disease (MMD) is rarely reported. We investigated serum metabolites in MMD and compared them with those of healthy controls (HCs) using a non-targeted gas chromatography–mass spectrometry (GC–MS) approach to identify metabolic biomarkers associated with MMD. Forty-one patients with MMD diagnosed by cerebral angiography and 58 HCs were recruited for our study. Comparative analyses (univariate, multivariate, correlation, heatmaps, receiver operating characteristi curves) were performed between MMD patients and HCs. Twenty-five discriminating serum metabolic biomarkers between MMD patients and HCs were identified. Compared with HCs, MMD patients had higher levels of phenol, 2-hydroxybutyric acid, L-isoleucine, L-serine, glycerol, pelargonic acid, L-methionine, myristic acid, pyroglutamic acid, palmitic acid, palmitoleic acid, stearic acid, octadecanamide, monoglyceride (MG) (16:0/0:0/0:0), and MG (0:0/18:0/0:0), and lower levels of L-alanine, L-valine, urea, succinic acid, L-phenylalanine, L-threonine, L-tyrosine, edetic acid, and oleamide. These metabolic biomarkers are involved in several pathways and are closely associated with the metabolism of amino acids, lipids, carbohydrates, and carbohydrate translation. A GC–MS-based metabolomics approach could be useful in the clinical diagnosis of MMD. The identified biomarkers may be helpful to develop an objective diagnostic method for MMD and improve our understanding of MMD pathogenesis.

## Introduction

Moyamoya disease (MMD) is a chronic, rare cerebrovascular-occlusive disease with a poor prognosis and considerable regional and racial differences (Kuroda and Houkin, 2008). The occurrence and development of MMD is multifactorial, and the underlying etiological and pathogenic mechanisms remain largely unclear (Huang et al., 2017). Most studies have focused on geographical distributions, sex predispositions, clinical manifestations, and age-specific characteristics (Okazaki et al., 2019; Sato et al., 2019). Basic research, including genomic and proteomic approaches, has been extensively conducted in the past 60 years. However, the physiological and metabolic mechanisms remain unclear.

China, especially southwest Shandong, has a large number of affected patients. MMD incidence peaks in children aged 5–9 years and adults aged 40–50 years with a female/male ratio of ∼2 and familial occurrence of 15% (Scott and Smith, 2009). The most common symptoms of MMD are headache, transient ischemic attack, infarction, and intracerebral hemorrhage. MMD often results in death, and early diagnosis is important. Cerebral angiography is the “gold standard” for diagnosis (Lee et al., 2013). However, cerebral angiography has flaws such as an inherent risk of anaphylaxis and nephropathy due to the use of contrast medium. The identification of new biomarkers to assist physicians in timely MMD diagnosis is needed to decrease morbidity and mortality.

Metabolomics is a promising approach that can identify metabolic patterns and disease-specific metabolic markers. This pivotal tool for biomarker discovery has improved the diagnosis of psychiatric illnesses (Cai et al., 2012), atherosclerosis (Chen et al., 2015), coronary artery disease (Turer et al., 2009), cerebral infarctions (Jung et al., 2011), and intracranial tumors (Pandey et al., 2017). However, information on metabolite profiles for MMD is limited. Only one metabolomic study on MMD patients has been conducted, and it focused on identifying metabolites in cerebrospinal fluid (CSF) using nuclear magnetic resonance (NMR) spectroscopy (Jeon et al., 2015). Therefore, more metabolomics studies are urgently needed to better understand the physiological and metabolic mechanisms involved in MMD.

We sought to elucidate the metabolic mechanisms underlying the occurrence and development of MMD. A GC–MS-based metabolomics approach coupled with uni- and multivariate analyses was employed to identify metabolic biomarkers associated with MMD. These results could help guide the development of an objective diagnostic method for MMD and provide insights into MMD pathogenesis.

## Materials and Methods

### Participants

Forty-one patients diagnosed by cerebral angiography as MMD and 58 HCs treated at the Jining First People’s Hospital, Jining Medical University between January 2018 and June 2019 were recruited for our study. Patients with MMD are often accompanied by basic diseases, such as hypertension, diabetes, and coronary artery disease. In order to make our research more rigorous, MMD patients with other types of diseases were not included in our study. In addition, the patients with MMD that we recruited were hospitalized patients who had a light diet 3 days before the operation. Similarly, 3 days’ diet of healthy people was recorded before we recruited to minimize the errors caused by these factors. Blood samples from all MMD patients were collected and subjected to centrifugation at 5,000 rpm for 10 min at room temperature and then stored at −80°C until analyses.

### Materials and Instruments

Heptadecanoic acid (purity: ≥98%; lot no. SLBX4162), as an internal standard (IS), was from Sigma-Aldrich (St Louis, MO, United States). Methanol was of chromatographic grade and purchased from Thermo Fisher Scientific (Waltham, MA, United States). Water was purchased from Hangzhou Wahaha Company (Hangzhou, China). Pyridine (lot no. C10486013) was from Shanghai Macklin Biochemical (Shanghai, China). *O*-Methyl hydroxylamine hydrochloride (purity, 98.0%; lot no. 542171) was obtained from J&K Scientific Industries (Ambala, India). *N*,*O*-Bis(trimethylsilyl)trifluoroacetamide with 1% of trimethylchlorosilane (BSTFA + 1% TMCS) (*v*/*v*; lot no. BCBZ4865) was purchased from Sigma-Aldrich.

### Preparation and Derivatization of Samples for GC–MS

Serum samples were processed according to the following procedure. First, 350 μl of methanol (containing 100 μg/ml of IS) was added to 100 μl of serum, vortexed, and centrifuged at 14,000 rpm for 10 min at 4°C. The supernatant was transferred to a 2-ml tube and evaporated to dryness at 37°C under the gentle flow of nitrogen gas. After the extracts had been dried, 80 μl of *O*-methyl hydroxylamine hydrochloride (15 mg/ml in pyridine) was added and mixed. The solution was incubated for 90 min at 70°C. Subsequently, 100 μl of BSTFA + 1% TMCS was added to each sample, followed by incubation for 60 min at 70°C. Samples were then detected by GC–MS.

### GC–MS

GC–MS was done on a 7890B GC system equipped with a 7000 C mass spectrometer. Separation was conducted on an HP-5MS fused-silica capillary column (30 m × 0.25 mm × 0.25 μm) with high-purity helium as the carrier gas at a constant flowrate of 1.0 ml/min. Each 1-μl aliquot of derivatized solution was run in split mode (50:1), with helium as the carrier gas and a front inlet purge flow of 3 ml/min; the gas flowrate was 1 ml/min. The GC temperature program was set to begin at 60°C for 4 min, increased to 300°C at 8°C/min, with a final 5-min maintenance at 300°C. The temperatures of the injection, transfer line, and ion source were 280, 250, and 230°C, respectively. Electron impact ionization (−70 eV) was used, with an acquisition rate of 20 spectra/s in the MS setting. MS detection was conducted by electrospray ionization (ESI) in full-scan mode from mass/charge (*m*/*z*) values of 50–800.

In general, 20 μl of each serum sample from MMD and HCs was obtained and then vortexed and mixed. Thus, this is the so-called quality control (QC) sample. Fifteen samples and five QCs were performed for 1 day (one batch), and 15 samples run randomized while the five QCs were distributed evenly among the 15 samples. At last, nine samples and five QCs were done. The peak area and retention time (RT) of the IS (heptadecanoic acid) were applied to evaluate the stability of the sample injection.

### Multivariate Statistical Analyses

GC–MS equipped with Unknowns Analysis and Agilent MassHunter Quantitative Analysis (for GC–MS) was used to process the GC data. This process enabled deconvolution, alignment, and data reduction to produce a list of m/z and RT pairs, with the corresponding intensities for all detected peaks from each data file in the dataset. The resulting table was exported into Excel^TM^ (Microsoft, Redmond, WA, United States), and the normalized peak area percentages were used as the percentage of corresponding intensities of each peak/total peak area. The resulting three-dimensional dataset including peak index (RT–m/z pairs), sample names (observations), and normalized peak area percentages was imported into SIMCA-P 14.0 (Umetrics, Umeå, Sweden) for statistical analyses. Center scaling, unit variance scaling, and pareto scaling are commonly used to perform the normalization data. In our paper, we adopted the pareto scaling. Too many missing values will cause difficulties for downstream analysis. There are several different methods for this purpose, such as replace by a small values, mean/median, k-nearest neighbor (KNN), probabilistic principal components analysis (PPCA), Bayesian PCA (BPCA) method, and singular value decomposition (SVD) method to impute the missing values (Kumar et al., 2017; Do et al., 2018). In our work, the default method replaces all the missing values with small values (the half of the minimum positive values in the original data) assuming to be the detection limit, and the data were not transformed.

A modified multicriteria assessment strategy was used to select variables. The assessment was used to reduce the number of variables and explore those that were most sensitive to interventions. The statistically significant threshold of variable importance in projection (VIP) values from the orthogonal partial least squares discriminant analysis (OPLS-DA) model was >1.0, and two-tailed Student’s *t* test differences of *p* < 0.05 were considered significant. “Fold change” was defined as the average mass response (area) ratio between two groups. Analyses of correlations, heatmaps, receiver operating characteristic (ROC) curves, and pathways were done using MetaboAnalyst 4.0.^1^ All results were shown in the “metabolome” view.

## Results

### Basic Participant Characteristics

Overall, 41 patients with MMD and 58 HCs were included in the analyses. Detailed information on the demographical and clinical characteristics of these participants is summarized in Table 1.

**TABLE 1:** Clinical characteristics of the participants.

		**No. of patients(%)**		
		**MMD**	**HCs**	***p* value**
	**Variables**	***n* = 41**	***n* = 58**	
Age	Mean ± SD, age (years)	42.94 ± 14.18	41.76 ± 11.06	0.6430
Sex, n(%)				0.9605
	Male	21(51.2)	30(51.7)	
	Female	20(48.8)	28(48.3)	
BMI	Mean ± SD, BMI (kg/m^2^)	23.48 ± 2.81	24.59 ± 3.91	0.1030
Smoking, *n*(%)	Smoking (%)	11(26.8)	15(25.9)	0.9142
Drinking, *n* (%)	Drinking (%)	8(19.5)	11(19.0)	0.9458
Type of onset, *n* (%)				NA
	Infarction	39(95.1)		
	Hemorrhage	2(4.9)		
Pathogenic site, *n*(%)				NA
	Bilateral	34(82.9)		
	Unilateral	7(17.1)		

MMD patients were not significantly different from HCs with regard to age, sex, body mass index (BMI), tobacco smoking, or alcohol consumption (all *p* > 0.05). The types of MMD onset were cerebral infarction (39 cases) and hemorrhage (2 cases). In addition, there were 34 cases of bilateral MMD and 7 cases of unilateral MMD.

### Metabolomics Analyses

**FIGURE 1:**
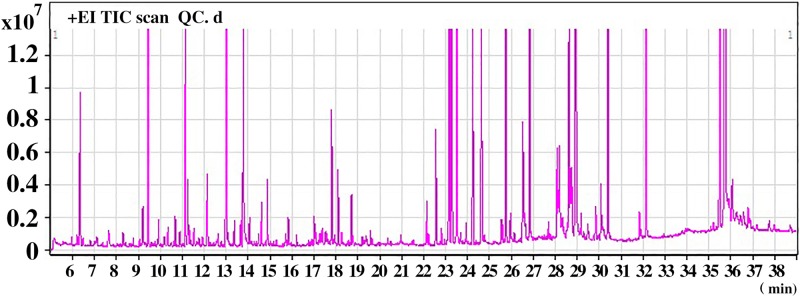
A representative gas chromatography–mass spectrometry (GC–MS) total ion chromatogram (TIC) of the quality control (QC) serum sample.

Representative GC–MS total ion current (TIC) chromatograms of the QC serum sample showed strong signals (Figure 1). In addition, the relative standard deviation (RSD) in intra- and interday of the peak area and retention time (RT) of the IS were <15%, indicating that the analytical instrument operated within acceptable standard variations.

**FIGURE 2:**
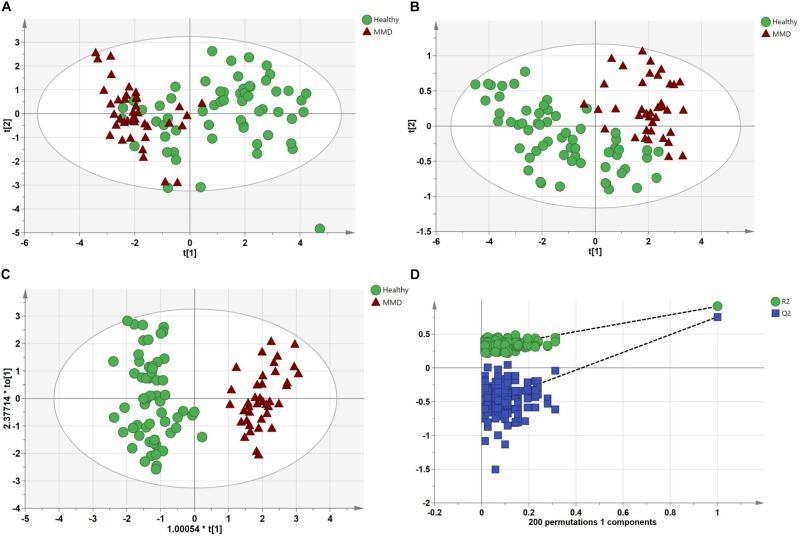
Multivariate statistical analysis between the control group and moyamoya disease (MMD) group. **(A)** Principal components analysis (PCA) scores plot; **(B)** partial least squares discriminant analysis (PLS-DA) scores plot; **(C)** orthogonal PLS-DA (OPLS-DA) scores plot; and **(D)** statistical validation of the OPLS-DA model through 200 × permutation testing.

After analyses of unknown compounds and quantitative analyses, 114 metabolites were identified in each serum sample and then used in the subsequent multivariate analysis. The PCA scores plot for HCs and MMD patients were *R*^2^*X* = 0.816, *Q*^2^ = 0.301. The pairwise PLS-DA score plots also suggested that the MMD patients were statistically different from the HCs: *R*^2^*X* = 0.579, *R*^2^*Y* = 0.836, and *Q*^2^ = 0.603. OPLS-DA analyses were carried out to maximize discrimination. The results suggested that this model was efficient and clearly separated the MMD patients and HCs (*R*^2^*X* = 0.823, *R*^2^*Y* = 0.913, and *Q*^2^ = 0.783). Values approaching 1.0 indicate a stable model with predictive reliability. Additionally, a permutation test with 200 iterations verified that the constructed OPLS-DA model was valid and not overfitted, as the original *R*^2^ and *Q*^2^ values to the right were significantly higher than the corresponding permutated values to the left: *R*^2^ = 0.289, *Q*^2^ = −0.556. They are shown in Figure 2.

### Identification of Potential Biomarkers

**FIGURE 3:**
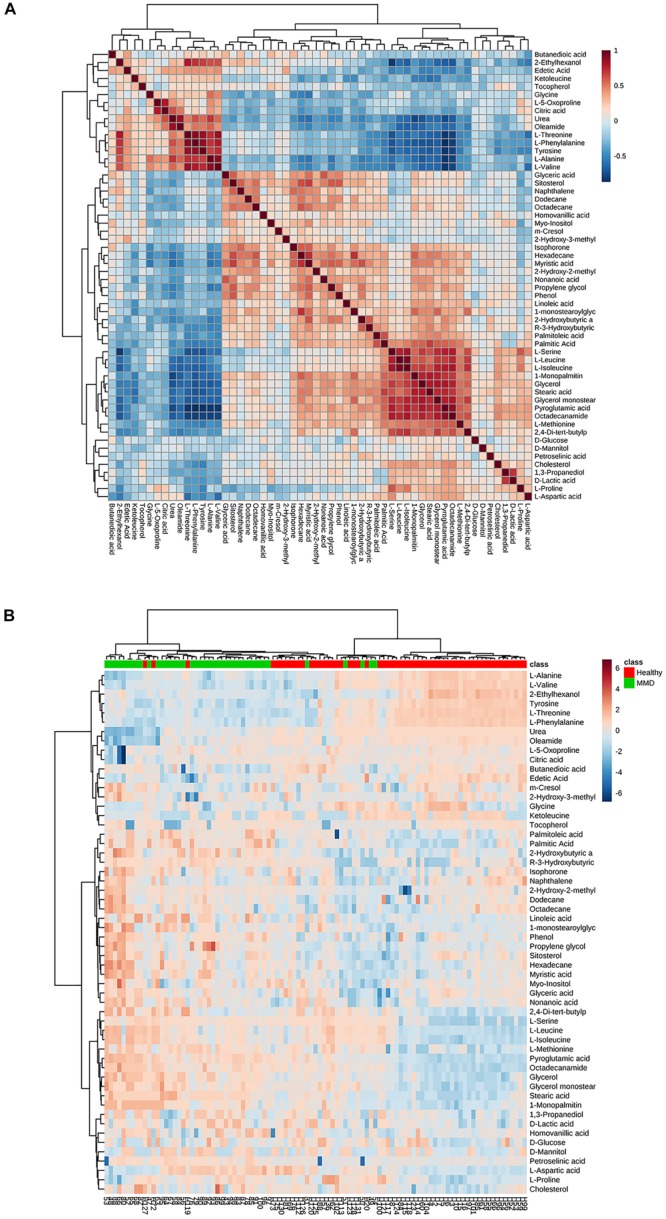
**(A)** Correlation analysis of the differential metabolites in moyamoya disease (MMD) patients and healthy controls (HCs). **(B)** Heat map for identified metabolites in MMD patients and HCs. The color of each section is proportional to the significance of change of metabolites (red, upregulated; blue, downregulated). Rows, samples; columns, metabolites.

Twenty-four metabolites to distinguish between the HCs and MMD patients were identified (VIP > 1, *p* < 0.05) (Table 2). Compared with HCs, patients with MMD were characterized by higher levels of phenol, 2-hydroxybutyric acid, L-isoleucine, L-serine, glycerol, pelargonic acid, L-methionine, pyroglutamic acid, myristic acid, palmitoleic acid, palmitic acid, stearic acid, octadecanamide, monoglyceride (MG) (16:0/0:0/0:0), and MG (0:0/18:0/0:0), as well as lower levels of L-alanine, L-valine, urea, succinic acid, L-threonine, L-phenylalanine, L-tyrosine, edetic acid, and oleamide. Their relationships were revealed by correlation analyses (Figure 3A). To better understand the metabolic differences between patients with MMD and HCs, data on identified metabolites were analyzed using clustering heatmaps. Even though sample clusters overlapped slightly, as shown in Figure 3B, most samples clearly grouped into two differentiated clusters, in agreement with OPLS analyses.

**TABLE 2:** List of assigned statistically significant metabolites between moyamoya disease (MMD) and healthy control (HC) group.

**Metabolites**	**HMDB**	**RT (min)**	**VIP**	***p* value**	**FDR**	**Fold change**
Phenol	HMDB0000228	9.144	1.144	8.00E-07	2.04E-06	1.601
L-alanine	HMDB0000161	10.344	1.414	2.27E-10	1.27E-09	0.235
2-hydroxybutyric acid	HMDB0000008	10.882	1.374	9.39E-10	4.78E-09	1.885
L-isoleucine	HMDB0000172	11.765	1.221	1.05E-07	3.45E-07	2.162
L-valine	HMDB0000883	12.632	1.470	2.80E-11	2.24E-10	0.127
Urea	HMDB0000294	13.030	1.432	1.17E-10	7.31E-10	0.382
L-serine	HMDB0000187	13.372	1.118	1.54E-06	3.76E-06	1.775
Glycerol	HMDB0000131	13.779	1.658	5.71E-15	1.60E-13	1.811
Succinic acid	HMDB0000254	14.359	1.158	5.63E-07	1.50E-06	0.666
Pelargonic acid	HMDB0000847	15.090	1.092	2.85E-06	6.64E-06	1.991
L-threonine	HMDB0000167	14.059	1.303	9.39E-09	4.04E-08	0.086
L-methionine	HMDB0000696	15.945	1.207	1.56E-07	4.61E-07	1.845
Pyroglutamic acid	HMDB0000267	17.317	1.678	1.96E-15	1.10E-13	4.578
L-phenylalanine	HMDB0000159	19.372	1.465	3.45E-11	2.41E-10	0.141
Myristic acid	HMDB0000806	22.216	1.041	9.07E-06	2.03E-05	1.314
L-tyrosine	HMDB0000158	22.811	1.476	2.25E-11	2.10E-10	0.228
Palmitoleic acid	HMDB0003229	26.512	1.165	4.67E-07	1.31E-06	1.336
Palmitic acid	HMDB0000220	24.630	1.507	6.21E-12	6.96E-11	1.437
Stearic acid	HMDB0000827	26.837	1.606	7.63E-14	1.42E-12	1.510
Edetic Acid	HMDB0015109	28.943	1.207	1.55E-07	4.61E-07	0.413
Octadecanamide	HMDB0034146	28.331	1.523	3.22E-12	4.50E-11	3.034
Oleamide	HMDB0002117	28.624	1.240	6.11E-08	2.28E-07	0.333
MG(16:0/0:0/0:0)	HMDB0011564	30.402	1.257	3.73E-08	1.49E-07	1.424
MG(0:0/18:0/0:0)	HMDB0011535	32.140	1.307	8.37E-09	3.91E-08	1.457

*FDR, false discovery rate; fold change, MDD/healthy; RT, retention time; VIP, variable influence on projection.*

### ROC Curve Analyses

**FIGURE 4:**
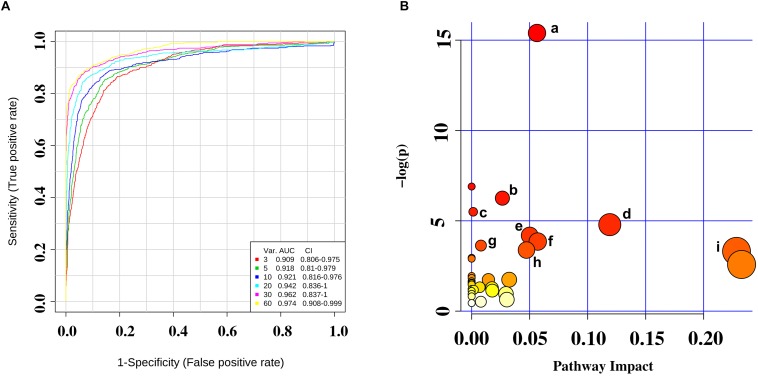
**(A)** Receiver operating characteristic (ROC) curves were for the sets of biomarker metabolites. **(B)** Summary of pathway analysis with MetaboAnalyst 4.0. (a) Aminoacyl-tRNA biosynthesis; (b) valine, leucine, and isoleucine biosynthesis; (c) propanoate metabolism; (d) phenylalanine metabolism; (e) cysteine and methionine metabolism; (f) alanine, aspartate, and glutamate metabolism; (g) phenylalanine, tyrosine, and tryptophan biosynthesis; (h) tyrosine metabolism; and (i) glycerolipid metabolism.

Further selection of potential indicator was performed by ROC analysis. Using different models, the value of the sensitivity and the area under the ROC curve (AUC) of these biomarker panels were both ≥0.8 (Figure 4A). An area of 1 represents a “perfect” test, so we obtained “good” efficiency for a clinical diagnosis for this set of metabolite biomarkers.

### Analyses of Metabolic Pathways

**FIGURE 5:**
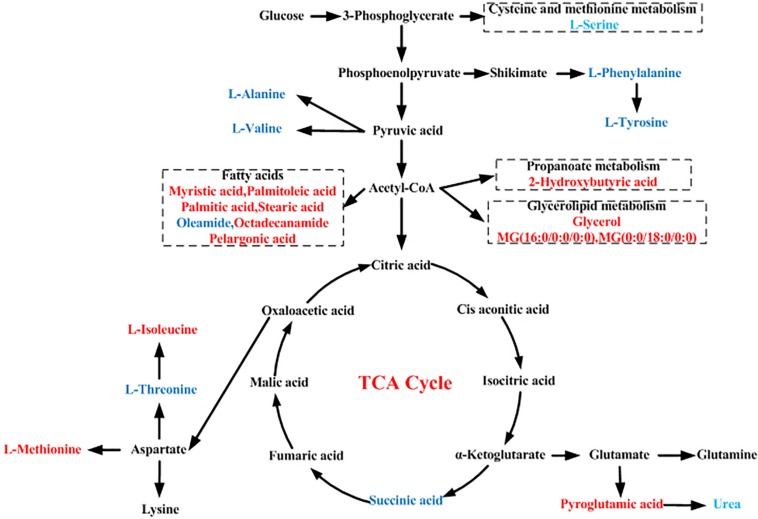
Schematic diagram of the proposed metabolic pathways in moyamoya disease (MMD) compared to healthy controls (HCs). Red and blue represent up- and downregulated metabolites, respectively.

We identified several pathways that may be significant (raw *p* < 0.5, impact >0) (Table 3). Nine pathways had the greatest significance: aminoacyl-tRNA biosynthesis: valine, leucine, and isoleucine biosynthesis; propanoate metabolism; phenylalanine metabolism; cysteine and methionine metabolism; alanine, aspartate, and glutamate metabolism; phenylalanine, tyrosine, and tryptophan biosynthesis; tyrosine metabolism; and glycerolipid metabolism (Figure 4B). The detailed results of the pathway analyses are shown in Table 3, with a summary shown in Figure 5.

**TABLE 3:** Results from pathway analysis from MetaboAnalyst 4.0.

**Pathway name**	**Total**	**Expected**	**Hits**	**Raw *p***	**Holm adjust**	**FDR**	**Impact**
Aminoacyl-tRNA biosynthesis	75	0.72	8	2.05E-07	1.64E-05	1.64E-05	5.63E-02
Valine, leucine, and isoleucine biosynthesis	27	0.26	3	1.92E-03	1.50E-01	5.12E-02	2.65E-02
Propanoate metabolism	35	0.33	3	4.09E-03	3.15E-01	8.18E-02	1.34E-03
Phenylalanine metabolism	45	0.43	3	8.33E-03	6.33E-01	1.33E-01	1.19E-01
Cysteine and methionine metabolism	56	0.53	3	1.52E-02	1.00E + 00	2.03E-01	5.00E-02
Alanine, aspartate, and glutamate metabolism	24	0.23	2	2.12E-02	1.00E + 00	2.43E-01	5.70E-02
Phenylalanine, tyrosine and tryptophan biosynthesis	27	0.26	2	2.65E-02	1.00E + 00	2.65E-01	8.00E-03
Tyrosine metabolism	76	0.73	3	3.40E-02	1.00E + 00	2.91E-01	4.72E-02
Glycerolipid metabolism	32	0.31	2	3.64E-02	1.00E + 00	2.91E-01	2.28E-01

*The Raw *p* is the original *p* value calculated from the enrichment analysis; the Holm *p* is the *p* value adjusted by Holm–Bonferroni method; the FDR *p* is the *p* value adjusted using false discovery rate.*

## Discussion

Moyamoya disease is a multifactorial disorder that likely presents unique pathophysiological profiles in each individual. Genetics, proteomics, and imaging have been used to discover markers for MMD (Araki et al., 2010; Maruwaka et al., 2015). However, there is no existing marker that could aid the diagnosis of MMD.

GC–MS-based metabolomics was applied to profile metabolic biomarkers in the serum of 41 MMD patients and 58 HCs. Our study is the first to identify serum biomarkers in MMD patients. Novel biomarkers may assist researchers in understanding MMD pathogenesis and provide new therapeutic strategies.

Owing to high separation efficiency, sensitivity, specificity, and throughput, as well as the development of various derivatization technologies, GC–MS has become important for the application of metabolomics. However, a single technology that can cover all metabolites in biological systems is lacking, and each technology has its own technical advantages and disadvantages. We discovered more metabolites than that in an NMR study of CSF samples of MMD patients (Jeon et al., 2015). There are several reasons for this diffidence, such as different matrix, different controls, and different instrument analysis. Twenty-four discriminating metabolites between HCs and MMD patients were identified using GC–MS analysis and used to establish a biomarker panel using logistic regression. The AUC was ≥0.8 for all samples (Figure 4A), indicating a “good” classifier of MMD patients and HCs. Furthermore, the mechanism of MMD could be obtained by assessing the pathways underlying these biomarkers.

As seen in Figure 3B, these discriminating metabolites were involved in nine significantly different pathways related to the metabolism of amino acids, lipids, carbohydrates, and translation of carbohydrates.

Overall, 20% of the human body is composed of amino acids and their metabolites that are basic substrates and regulators in many metabolic pathways. Amino acid levels in patients with various diseases often differ from those of healthy individuals. Alterations in plasma concentrations of amino acids have been reported in liver fibrosis, non-small-cell lung cancer, aortic dissection, first-episode psychosis, and type-2 diabetes mellitus in patients with coronary artery disease (Yang et al., 2013; Leppik et al., 2018).

We found that levels of a panel of amino acids (L-alanine, L-isoleucine, L-valine, L-serine, L-threonine, L-methionine, L-phenylalanine, and L-tyrosine) were significantly different in MMD patients relative to HCs. Levels of L-alanine, L-valine, L-threonine, L-phenylalanine, and L-tyrosine were decreased in MMD patients, whereas L-isoleucine, L-serine, and L-methionine levels were increased. Such alterations of several amino acids suggest that amino acid metabolism is disturbed in MMD.

The synthesis of L-alanine is derived from pyruvate by alanine aminotransferase directly involving in gluconeogenesis and the alanine–glucose cycle and regulates glucose metabolism (Chen et al., 2017). Like gamma-aminobutyric acid, taurine, and glycine, L-alanine is an inhibitory neurotransmitter in the brain and is involved in lymphocyte reproduction and immunity. L-Alanine is disturbed in serum of MMD patients, but the association between L-alanine and MMD has not been investigated. Our study did not elucidate how altered levels of L-alanine affect MMD development; more studies are needed. L-Isoleucine and L-valine are branched chain amino acids (BCCAs). They are critical to human life and are particularly involved in stress, energy generation, and muscle metabolism (Zheng et al., 2017; Zhenyukh et al., 2017). The biosynthesis of L-isoleucine is initiated by the L-threonine deaminase reaction, whereas the pathway toward L-valine starts from pyruvate. Acetohydroxyacid synthase, ketol acid reductoisomerase (KARI), dihydroxyacid dehydratase (DHAD), and branched chain amino transferase (BCAT) were the four enzymes operating in L-isoleucine and L-valine biosynthesis (Galili et al., 2016). Abnormal changes in L-isoleucine and L-valine levels have been documented in first-episode psychosis (Leppik et al., 2018). Our study suggested that abnormal changes in L-isoleucine and L-valine levels are also associated with MMD.

Like glutamate, aspartate, and glycine, L-serine can act as an excitatory and inhibitory neurotransmitter (Yu et al., 2017; Martin et al., 2018). As seen in Figure 5, phosphoenolpyruvate (PEP) is one of the precursors to the shikimate pathway; in addition, PEP is also derived from oxaloacetate by phosphoenolpyruvate carboxykinase (PEPCK) in glycolysis; thus, they are linked to each other. L-Serine may be derived from the biosynthesis of the glycolytic intermediate 3-phosphoglycerate, which participates in cell proliferation and is necessary for specific functions in the central nervous system. Altered levels of L-serine in patients with psychiatric disorders underscore the amino acid’s importance in brain development and function. Thus, L-serine level alterations may be involved in MMD pathogenesis by affecting brain development and function. L-Threonine is an essential amino acid in humans, and severe deficiency causes neurological dysfunction (Liu et al., 2010). In our study, L-threonine level was lower in serum of MMD patients compared to HCs, indicating that L-threonine was altered in serum of MMD patients. Alterations in the ratio of L-tyrosine and L-phenylalanine levels could be a sign of compromised function of the dopaminergic system, which may be partly associated with MMD pathogenesis.

Collectively, our findings indicate that altered levels of amino acids could be linked to MMD. The precise mechanism by which amino acid levels influence the genesis and development of MMD should be investigated further. Pyroglutamate is converted to urea over glutamate and allantoin reactions; additionally, L-arginine could be converted to urea via urea cycle. Altered levels of pyroglutamate and urea were found in MMD. A panel of fatty acids (succinic acid, myristic acid, palmitoleic acid, palmitic acid, stearic acid, oleamide, octadecanamide, and pelargonic acid) could be used to distinguish between MMD patients and HCs. Succinic acid is an intermediate of the tricarboxylic acid (TCA) cycle (Bechthold et al., 2010; Lauritzen et al., 2014). Levels of succinic acid were significantly decreased in MMD patients compared with HCs. Lower levels of TCA-cycle intermediates may indicate alterations in the cycle. The TCA cycle is the core of cellular respiratory machinery and produces energy to power manufacture of compounds needed to defend against oxidative stress. Oxidation of fatty acids in mitochondria is responsible for approximately half of the total amount of adenosine triphosphate generated. Fatty acids also serve as the “building blocks” of cellular membranes after their esterification into phospholipids and are involved in signal transduction. Altered levels of fatty acids would lead to decreased energy production. However, whether such lower energy levels are associated with an increased risk of MMD is unknown.

The OPLS-DA method was used to analyze metabolic features and maximize discrimination between classes of compounds (Westerhuis et al., 2010). This approach reduced the effects of variability of non-relevant metabolites and helped identify serum metabolites contributing to differences between MMD patients and HCs. The reliability of OPLS-DA results was confirmed by correlation analyses and heatmaps.

Our study had two main limitations. First, MMD is an uncommon disease. We recruited 41 MMD patients, which may have introduced selection bias because of interindividual differences. Second, we only used a metabolomics approach, and the possibility that single-omic data restricted interpretation of our results cannot be excluded. Proteomics and genomics analyses are needed to confirm our findings.

In summary, we used a GC–MS platform to characterize the metabolic profiles of serum from MMD patients. Our analysis revealed important candidate metabolic biomarkers for MMD. Verification and validation studies with larger independent samples are necessary to demonstrate the utility of these metabolites as potential disease markers and to elucidate the pathophysiological mechanisms underlying MMD.

## Data Availability Statement

The raw data supporting the conclusions of this manuscript will be made available by the authors, without undue reservation, to any qualified researcher.

## Ethics Statement

Our work was carried out according to the Declaration of Helsinki. All participants provided written informed consent to allow use of their samples in these analyses, and our study protocol was reviewed and approved by the Medical Ethics Committee of the Jining First People’s Hospital (Jining, China) (No. 20170021).

## Author Contributions

PJ and CC conception and design. CW, JZ, and WH acquisition of the data. CG and PJ analysis and interpretation of the data. CG drafting the manuscript. PJ and FJ critically revising the manuscript. CG, PJ, CC, and FJ reviewed submitted version of manuscript. PJ approved the final version of the manuscript on behalf of all authors. CG, DC, and YG statistical analysis. PJ study supervision.

## Conflict of Interest

The authors declare that the research was conducted in the absence of any commercial or financial relationships that could be construed as a potential conflict of interest.

## Footnotes

1 www.metaboanalyst.ca/
